# The independent directors' Communist Party of China member status and firms' ESG performance: Evidence from Chinese listed firms

**DOI:** 10.1016/j.heliyon.2024.e36789

**Published:** 2024-08-28

**Authors:** Fang Jia, Xuerong Li, Yan Gao

**Affiliations:** a Food Safety Research Center, Wuhan Polytechnic University, Key Research Institute of Humanities and Social Sciences of Hubei Province, 36 Huanhu Middle Road, Wuhan, 430048, Hubei, China; b School of Management, Wuhan Polytechnic University, 36 Huanhu Middle Road, Wuhan, 430048, Hubei, China; c Yiwu Internation Trade School, J number 180, Chengbei Road, Yiwu, 322000, Zhejiang, China

**Keywords:** Independent directors 1, CPC2, ESG3

## Abstract

Under the leadership of the Communist Party of China (CPC), this study aims to explore the relationship between independent directors who are CPC members and firms' ESG (environmental, social, and governance) performance. We use a sample of 30,629 A-share firms in China for the period 2012–2021. Our main results show that CPC membership among independent directors can significantly improve firms' ESG performance, with board meetings serving as an important mechanism for this effect. We also examine the impact of CPC membership on external supervision, attention levels, “full and strict governance over the Party”, and ownership nature. The influence of independent directors' CPC membership on firms' ESG performance is greater in companies lacking external supervision, those with higher attention levels, and in state-owned enterprises (SOEs).

## 1 Introduction

ESG (Environmental, Social, and Governance) has widely received much attention from various stakeholders including suppliers, customers, regulators, institutional investors, and managers. According to the stakeholder theory, various resources supported by stakeholders are indispensable for firms' operation, so shareholders attach their attitudes to ESG for long-term value [31], [49]. Nonetheless, some research indicates that firms' ESG performance currently lowers its market value, which hinders management from actively enhancing a firms' ESG performance out of self-interest [8]. Additionally, according to stakeholder theory, independent directors are more inclined to prioritize the welfare of stakeholders [57], [20]. Agarwala, Pareek, and Sahu [1] has also discovered that independent directors are more engaged in corporate social responsibility (CSR) and stakeholder attention for long-term benefits. Furthermore, independent directors are more likely to oppose the CEO and stand up for investor interests. Because they do not face the same employment or advancement concerns as internal directors.

Communist Party of China (CPC) and the government regard harmony between the economy and the environment as an important goal. The impact of political connections on firms has received widespread attention, but the existing evidence on how CPC affects firms' ESG performance is not unanimous. Some studies indicate that CPC membership has a huge impact on promoting the acquisition of bank loans [35], hindering the firm's international expansion [43], easing agency problems, and preventing firms' overinvestment [37]. Scholars have demonstrated that CPC members exert various effects, including human capital, signaling, political and social capital, as well as negative rumor effects. These factors collectively contribute to their significant impact on the development of firms [40], [45], [64]. CPC membership China signifies a measure of political status and, crucially, denotes a significant affiliation with the sole ruling party in China. As of December 2021, the membership count of the Communist Party of China has reached 97712000, and the Communist Party of China now has about 4,936,000 grass-roots organizations. Worthwhile, according to the “2021 Statistical Bulletin of the Communist Party of China”, on 31 December 2021, a total of about 1,703,000 legal entities of non-SOE had established Party organizations nationwide, the Communist Party of China has a great influence on firms' development. One stream of literature finds that firms with political connections actively engage in ESG activities for firm reputation and regulatory restraints [49]. Another stream of the literature suggests that political connections are often regarded as a protective umbrella to help firms away from costly compliance measures, further reducing the motivation to engage in ESG [46].

Practically, hiring managers with political connections is a common way for listed firms to obtain various resources [60], [63]. China's political system determines that the CPC party branches 1(also known as the Party Committee) play a critical role in Chinese firms. The higher proportion of CPC) member status in a firm, which means stronger political connections. Given the absolute dominance of the Communist Party in China's political system, party membership implies an important political affiliation. Consequently, government authorities may potentially exhibit preferential treatment towards party members when assigning jobs [45]. Some literature shows that CEOs with CPC member status [61], entrepreneurs with Party membership [64], and chairmen with CPC member status [63], could bring resources that affect firms' decision-making. Besides, according to the upper echelons theory, firms' decisions are a reflection of their executives' perceptions of the environment and the level of attention they allocate to specific environmental factors [24].

Agency conflict is caused by this incentive differential, which can be reduced by good corporate governance, especially a strong board of directors [54], [7]. Effective boards align the interests of management and shareholders by providing oversight, guidance, and monitoring. Separation of ownership and management raises agency issues, firms start to introduce independent directors to mitigate the agency issues. CSRC issued guidelines governing the role of independent directors in listed firms and also set out requirements for the background of independent directors^2^, and studies show that different backgrounds of independent directors influence the board behaviors and the output of the firms. Therefore, under the distinctive feature of the corporate governance system in China, independent directors' CPC member status has an important influence on corporate governance. According to the stakeholder theory, corporate disclosure is used by the management as a tool to provide information for the various stakeholders, so conflict resolution serves as a motivator for firms to engage in CSR initiatives. A lot of research shows how political connections affect firms' financial performance from various perspectives [38], [58], but there is little research on how independent directors with CPC member status affect firms' ESG performance.

Independent directors who may work for several different firms typically have better access to party policies and stakeholders' ESG preferences, which improves their advice and competency, and facilitates easier communication across the firms [39], [30]. Such a network relationship contributes to the more transparent generation of party culture and ESG information, facilitating the supervision of managers at all levels, and preventing them from abusing their authority to have political rent-seeking or corporate ‘greenwashing’ behavior. Therefore, independent directors who are party members are more capable of planning, deploying, and implementing the construction of corporate culture in conjunction with party building. The CSCR announced a revised version of the “Guidelines for Corporate Governance of Listed Firms” in 2018, introducing the ESG concept currently promoted in the international capital market, strengthening the leading role of listed firms in environmental protection and social responsibility, and establishing a basic framework for ESG information disclosure. Compared to internal directors, independent directors are sensitive to outside information about firms' development. Affected by CPC member status, independent directors who are CPC members consider guidelines when monitoring the firms' behavior or providing information for decision-making.

In this study, we choose Chinese listed firms from 2012 to 2021 as samples to investigate the relationship between independent directors with CPC member status and firms' ESG performance. The results find that independent directors with CPC member status can positively improve firms' ESG performance. Then, we conduct a set of robustness checks on our empirical findings. We reexamine our CPC-embeddedness effect using an alternative definition of the independent variable, alternative definition of the independent variable, lagged explained variables, Heckman Two-Stages, IV-2SLS, and DID, the results were the same and confirmed the results of the previous OLS regression. Additionally, we investigate the mechanism, moderator effect, and motives. Finally, we discuss CPC-embeddedness in other ways, such as external supervision, attention level, “full and strict governance over the Party”, and ownership nature.

Our research provides several key contributions to the existing literature. First, this paper enriches the literature on the influencing factors of ESG in Chinese listed firms. There exists considerable research mainly documents the influencing factors of firms' ESG performance from other views of firms, such as financial constraints, free cash flow [6], CEOs' political status [61], and chairman's political status [63]. This paper investigates how independent directors with political status impact ESG. Specifically, the independent directors with CPC member status improve the firms' ESG performance. Second, this paper enriches the research on the upper echelon theory in China from the perspective of independent directors' political characteristics, CPC members lead independent directors to positively perform to the best of their abilities and influence their subordinates. Third, we also find two unexpected results. On the one hand, independent directors' CPC member status is unable to play the functional role of their governance in firms' ESG performance when the number of independent directors concurrently holds independent directors in other firms, the main reason is that concurrent posts will distract the attention of the independent directors. On the other hand, the independent directors' CPC member status has the supervisory alternative role.

## 2 Literature review and hypothesis development

### 2.1 Literature review

Environmental, social, and governance (ESG) has received growing attention from investors, customers, executives, and other stakeholders in recent years. Some empirical show that ESG/CSR activities are not in the interests of stakeholders but rather an outcome of agency problems [61], [13], they suggest that any benefit to stakeholders from social responsibility comes at the direct expense of firm value, managers do not actively advocate firm ESG activities for their benefit. It is crucial for independent directors to engage actively in the governance of listed companies, serving as both supervisors and advisors to safeguard the legitimate rights and interests of stakeholders [17]. Meanwhile, Sun et al. [56] found higher ESG performance are less likely to engage in earnings management, so the firms' ESG performance as an important signal for stakeholders.

According to the upper echelons theory, managers are driven by tasks, and their distinct personal attributes can enhance organizational performance by influencing decision-making [7], [48]. This theory acknowledges that the decisions made by top managers can greatly affect organizational outcomes, and these decisions are influenced by the managers' individual characteristics. Some researches shown that managerial myopia the significantly reduced the firms' ESG performance [56], [16]. The inclusion of independent directors on the board encourages organizations to be more vigilant against managerial opportunism and holds executives accountable for their actions. This enhanced supervision strengthens managerial accountability, motivating them to exert their utmost effort and positively influence their subordinates. Numerous researchers find that the effects of corporate governance are based on independent directors' characteristics or backgrounds, such as education level [33], [4], gender [22], political connection [68], [36], and expertise [47]. Summing up, the independent director's attributes can influence the management in actively participating in ESG activities.

Promoting the construction of harmony between the economy and the environment is a strategic initiative of the Communist Party of China and the government for governance in the new era. At the Fifth Plenary Session of the 18th CPC Central Committee in 2015, the meeting introduced a new sustainable development philosophy including innovation, coordination, green, openness, and sharing. Sustainable development gradually became a national goal, and the importance of ESG is arising [52]. Under the leadership background of the Communist Party of China, the party is embedded in corporate governance [65]. The study by Hu et al. [29] shows that political connections play an important role in firms' ESG performance, according to several studies demonstrating that firm, management, or country-wide factors affect firms' ESG performance. The system of “dual entry and cross-appointment” has far-reaching implications for the Chinese Communist Party's control over firms. This system ensures that managers adhere to the Party's strategies, guidelines, and policies during their production and operational processes [66]. The ideologies of revolution and reform in the Party are formulated to embed the CPC's beliefs into people's consciousness and to retain consensus. Xiaorong Li and Ma [62] and He, Du, and Yu [28] have found that with the continuous strengthening of Party building activities in China, Party committees within enterprises exert a substantial influence on operational decision-making and enhance governance efficiency. This is reflected in reducing agency costs, suppressing shareholder tunneling behavior, protecting firms' interests, and inhibiting the expansion of salary differentiation between executives and ordinary employees [52], which directly impact the firms' investment in ESG.

Moreover, the principle of ‘two-way entry and cross-posting’ is firmly ingrained within firms. The “two-way entry” refers to members of the Party Committee being integrated into the board of directors, the board of supervisors, and the senior management team through statutory procedures. In contrast, Party members on the board of supervisors and in senior management roles simultaneously assume positions within the Party Committee, in accordance with relevant regulations. Wang, Tian, and Dang [61] demonstrated that the executive's CPC member status significantly influences their behaviors. The term “cross-posting” means that the secretary of the Party Committee and the independent directors are appointed by the same person. Independent director is a vital factor in corporate governance. According to the upper-echelon theory, independent directors' backgrounds or characteristics have a great impact on corporate governance. For example, Hu et al. [29] find independent directors with financial expertise can effectively reduce the level of firm earnings management. Studies also show that independent directors with political linkages have an impact on firms' financial performance [13], innovation [14], and institutional norms [50]. Therefore, the political preference of independent directors affects firm performance.

While members of the CPC are effective channels for firms to communicate with the government. In this way, the government may prioritize firms with good relations with the government when implementing ESG/CSR policies [15], [23], which means that the members of CPC can bring more external resource support for firms, such as tax incentive, government subsidies, bank loans, etc. These resources may also help firms realize the regulatory mandates and public policy decisions [41]. Several studies have shown that the party member status of the top management team influences their behaviors, Xie et al. [63] found that chairmen who have CPC member status can alleviate poverty, Wang, Tian, and Dang [61] also found executives' CPC member status can improve firms' financial performance, and Yan and Xu [64] found the entrepreneur with CPC member status are able to promote firms to develop social responsibility. In addition, independent directors under the pressure of CPC's reputation will pay more attention to government regulations and industry rules [38].

As China does not currently have ESG disclosure as a mandatory regulatory requirement, which makes ESG disclosure not a good situation for firms in China, the attitude of corporate management or governance towards ESG. Corporate governance strategies have been significantly influenced by independent directors, who fulfill supervisory and advisory roles. Similarly, CPC members actively participate in party activities, enhancing their political awareness and executive capabilities through education and training. Party branches within firms, by instilling ideological values in management, significantly advance environmental corporate social responsibility (ECSR) among Chinese listed firms [55]. Independent directors with CPC membership status will therefore incorporate the Party's emphasis on selfless dedication and wholehearted service to the people into corporate governance. The government derives its authority from the law and the constitution, and the rule of law is at the core of its operations. As a result, the government is not as binding on firms' ESG performance.

### 2.2 Hypothesis development

Based on agency theory and stakeholder theory, independent directors are more willing to supervise managerial opportunism or provide ESG information to managers to alleviate conflict of interest [57], [20]. Political parties systematically integrate corporate sustainability and governance practices to mitigate policy uncertainty. Independent directors as the bridge between the outside and inside of a firm, they can effectively pass on policy messages. Chinese communists have an innate sensitivity to Party policies. Party organizations' “Pre-discussion” decision-making mechanism can significantly promote firms' ESG performance. Independent directors with CPC member status leverage their supervisory and advisory roles to transmit new Party political information into the internal governance of firms.

China, a socialist nation under the leadership of the Communist Party of China (CPC), possesses a distinct political and cultural heritage. As the bearers of communist culture, Communist Party members facilitate the exploration of the influence of this culture on corporate social responsibility. Members of the Communist Party who are independent directors undergo regular party education and training. This process embeds communist cultural values deep within their ideology and personal values. Due to these profound values, Communist Party members serving as independent directors are able to develop value-based judgments regarding their responsibilities and actions in daily business activities. Consequently, independent directors who are Communist Party members are better positioned to implement the Party's guidelines and policies, thereby steering firms towards a proactive fulfillment of social responsibility.

Some studies show that CPC membership influences their behaviors. The CPC membership of chairman in private firms significantly improve the firm's commitment to and investment in poverty alleviation efforts [63]. Wang et al. [58] investigate how CPC participates in corporate governance, and find the executive's CPC member status can significantly increase firm value. CPC control, in terms of having a CPC member as a director, supervisor, or senior executive, can improve investment efficiency [62]. In addition, the system of “dual entry and cross-appointment” was introduced into firms, it can effectively reduce the immoral of executives. On one hand, executives who are Communist Party members tend to place greater emphasis on their social reputation and are more focused on political advancement than on salary. On the other hand, the CPC adheres to the principle of ‘Party supervision of cadres,’ requiring executives to willingly accept oversight from the Party.

Resource dependence theory posits that firms may gain valuable resources and protection through CPC membership, which influences their engagement with ESG concerns. Additionally, studies indicate that businesses acquiring more scarce resources typically increase their ESG and CSR initiatives [25]. Furthermore, the process of becoming a CPC member typically involves 3-5 years of training. According to the theory of ideological imprinting, the Party culture instills values of ‘serving the people’ and ‘contributing to society’ deeply in individuals, leading to a stronger alignment with ESG/CSR principles. As a result, independent directors who are CPC members implement the Party's objectives, further guiding firms to actively engage in CSR initiatives [64], [37].

In summary, we argue that independent directors' CPC member status may improve firms' ESG engagement. These considerations lead to the following hypothesis:

H1a: The independent directors with CPC membership will improve firms' ESG performance.

However, independent directors with CPC membership can also induce a firm's lower ESG performance. Party members serve as crucial intermediaries, facilitating the government's communication of public demands to enterprises and influencing their environmental decisions. Some literature suggests that “bridges” may be viewed as a protective umbrella to evade costly compliance measures, which would reduce the motivation to engage in ESG [41]. The political affiliations and strategic political resources of independent directors with government official positions facilitate their rent-seeking motivation, the political status creates an advantage for firms [68]. Independent directors as the bridges between firms and government, firms may use the “umbrella” to avoid engaging in ESG-related activities and even undermine ESG goals without facing government penalties. Such CSR activities as tools for actors' political agendas and personal goals are not conducive to improving stakeholders' welfare or creating social value. Moreover, the establishment of CPC party organizations by non-SOEs may be more likely to have good relations with the government [23]. Governments may inadvertently enable or overlook excessive emissions by polluting firms through the relaxation of environmental regulations and intervention in corporate governance, potentially leading to a disregard for firms' ESG performance. Some studies reveal that CPC member status is less inclined to prioritize financial performance [42], [59]. Practically, many independent directors are contacted and employed by management or controlling shareholders, and the salaries and benefits of independent directors are essentially determined by management or controlling shareholders. Independent directors will inevitably be dependent on and subject to management or controlling shareholders in the performance of their duties. Under the pressure of corporate profit maximization, independent directors may be lax in regulating ESG activities, or even refrain from ESG activities in order to reduce firms' costs.

Independent directors may utilize their CPC membership as a “protective umbrella” to focus on corporate profit maximization goals. Consequently, firms' ESG activities are likely to decrease. This situation could lead to independent directors not enhancing, or even potentially reducing, a firm's engagement in ESG practices.

In summary, we argue that independent directors' CPC membership may enhance firms' ESG engagement. Thus, we propose the following hypothesis:

H1b: The independent directors with CPC member status do not enhance firms' ESG performance.

## 3 Data and variables

In this section, we describe the key variables used in our paper and the sample constructions.

### 3.1 Data

Our initial sample is selected from A-share listed companies from 2012 to 2021. We obtain the data of firms' ESG performance from the Bloomberg ESG database. Amid the increasing societal focus on ESG, various rating systems have emerged in China, such as the SynTao Green Finance Rating, the Social Value Investment Alliance Rating, and Sino-Securities Index Rating. The Bloomberg ESG database offers a comprehensive range of information sources and exhibits a high degree of objectivity [18], [31]. The 18th National Congress of the Communist Party of China was held on November 8, 2012, and then Rule No. 18 was issued 3, which is why we focused our initial sample on this period.

Bloomberg's ESG rating is based on recommendations from the Global Reporting Initiative and ratings are frequently cited in academic research, as evidenced by studies. Bloomberg ESG rating system covers the firms' information on the environment, society, and governance from many channels, such as government announcements, corporate annual reports, and media reports. Importantly, the rating system focuses on the participation of firms' ESG practice, rather than merely quantifying ESG information.

We obtain the data of the CPC member status for independent directors, party branches, and other financial data of firms from the China Stock Market & Accounting Research (CSMAR) Database.

To ensure the scientificity and accuracy of the research results, according to the research characteristics of this paper, the original sample is treated according to the following criteria: (1) Firms from the financial, insurance, and real estate industries are removed, we consider that accounting standards for these sectors are not the same as for ordinary businesses. (2) Firms warned of other significant risks, also named as special treatment (ST). Delisted firms are removed due to differences in limitations on price rises and falls. (3) Observations with incomplete data were excluded from the analysis. Additionally, to mitigate the influence of outliers, all variables were subjected to winsorization at 1 and 99% levels. Our final sample consists of 30629 observations of 5138 firms from 2012 to 2021.

### 3.2 Variables

The Bloomberg's ESG rating provides the evaluation of firms' ESG performance by scores which range from 0 (for firms that do not disclose ESG) to 100 (for firms that disclose all ESG data recognized by Bloomberg).

Following existing studies [36], [63], to more accurately measure the impact of CPC on independent directors, we constructed the following multidimensional variable system. We use the dummy variable IDparty as the independent directors' CPC member status. If the independent director is a member of the CPC, the value of IDparty is 1; otherwise, is 0. Specifically, We search through the resumes of corporate directors and supervisors, and if the keyword ‘party member’ appears, we assume that he/she is a party member and set the dummy variable to 1, otherwise, it is 0. Furthermore, we cross-multiply the dummy variable of IDparty with independent director to generate the dummy variable of Party member independent director.

We also controlled for other firm-specific characteristics, such as firms' size, profitability, financial leverage, and so on. Comprehensive descriptions and definitions of the variables used in this study are available in Table 1.

**Table 1:** Definition of variables.

Type of variable	Variable name	Symbol	Definition
Dependent variables	ESG performance	ESG	ESG Composite Indices in the Bloomberg database.
Independent variables	Independent directors' CPC	IDparty	1 if independent director is CPC member, else 0.
Moderating variables	Administrative linkage	Consultative	1 if director is NPC deputy and CPPCC member, else 0.
	Association linkage	Polity	1 if director is FICCI member, else 0.
Mediating variable	Board meetings	BM	Frequency of board meetings.
Control variables	Firm size	Size	Logarithm of total assets.
	Profitability	Roa	Net profit divided by total assets.
	Asset capacity	TobinQ	Market value of a bank divided by the book value of assets.
	Financial leverage	Lev	Liabilities divided by total assets.
	Operating cash flow	Ocf	Net cash flow from operating activities divided by total assets.
	Enterprise age	Age	Time since establishment of firm.
	Board size	Board	Number of directors on board.
	Proportion of independent directors	IDratio	Percentage of independent directors on the board
	Shareholding proportion of largest shareholder	Top1	Percentage shareholding of largest shareholder.
	Dual roles	IsDuality	1 if chairman and CEO roles held by one person, else 0.
	Attention level	Attention	the number of concurrent independent directors' CPC appointments.
	External supervision	Big4	1 if the firms are supervised by Big Four accounting firms, else 0.
	Property nature	SOEs	1 if the firm of property nature is state-owned, else 0.

## 4 Empirical results and analysis

### 4.1 Empirical model

#### 4.1.1 Baseline regression model

To capture the effect of independent directors' CPC member status on firms' ESG performance, we construct the regression models as follows:

(1) $$ESG_{i,t}=\alpha_{0}+\vartheta_{2}IDcpc_{i,t}+\beta_{2}controls_{i,t}+Ind_{i}+Year_{t}+\varepsilon_{i,t}$$

In the above Equation (1), $ESG_{i,t}$ is the score of ESG for firm i in year t. $IDcpc_{i,t}$ is the independent variable, and we utilize IDparty (CPC member status of independent directors). $\varepsilon_{i,t}$ represents the random error term. Control variables include a range of firm characteristics that may influence firms' ESG performance, such as firm size (Size), profitability (Roe), financial leverage (Lev), operating cash flow (Ocf), firms age (Age), asset capacity (TobinQ), TThe size of Board (Board), the precentage of independent directors on the board (IDratio), the shareholding proportion of the largest shareholder (Top1), the two positions are in one (IsDuality), and the number of concurrent independent directors' CPC appointments (Attention), External supervision (Big4), and the number of concurrent independent directors' CPC appointments (Attention), External supervision (Big4), and property nature (SOEs). We also add industry and year-fixed effects to the regression.

### 4.2 Analysis of the results

#### 4.2.1 Descriptive results

Table 2 provides the descriptive statistics for the all variables. The average value of the ESG scores is 28.098, with ESG scores from 11.157 to 51.413, this suggests that there is considerable variation in the distribution of the ESG performance during 2012-2021 in China. The standard deviation of ESG is 7.784. The average of IDparty is 0.014, suggesting that 1.4% of the independent directors in the sample firms are members of the Communist Party of China. Other control variables align with prior research.

**Table 2:** Descriptive Statistics of Variables.

Variable	Obs	Mean	Std. Dev.	Min	Max
ESG pengbo	30629	28.05	7.784	11.157	51.413
IDparty	30629	0.014	0.118	0	1
Polity	30629	0.012	0.108	0	1
Consultative	30629	0.027	0.163	0	1
Attention	30629	0.898	0.302	0	1
Big4	30629	1.928	0.259	1	2
SOEs	30629	0.102	0.303	0	1
Policy	30629	0.168	0.374	0	1
Top1	30629	31.57	14.7	7.12	73.67
IDratio	30629	0.378	0.063	0.25	0.571
IsDuality	30629	0.018	0.134	0	1
Roa	30629	0.039	1.214	-56.538	0.874
Size	30629	22.294	1.175	19.732	25.836
Lev	30629	0.45	0.198	0.008	0.995
TobinQ	30629	0.55	0.197	0.116	0.945
Board	30629	8.76	1.731	4	18
Age	30629	13.44	6.912	2	31
Ocf	30629	0.147	0.111	0.001	0.849

* This table shows the descriptive statistics for variables based on a sample of A-share listed firms in China covering the period from 2012 to 2021. All variables are winsorized at the 1% and 99% levels to mitigate outlier effects. ESG represents a firm performance score sourced from Bloomberg. IDparty is a binary variable indicating the independent directors with CPC member status. The table includes counts of observations, means, standard deviations, minimums, and maximums for each variable. Definitions for other variables are provided in Table 1.

#### 4.2.2 Baseline regression results

We conducted a series of regression analyses to examine the effects of independent directors' CPC member status on firms' ESG performance. Table 3 displays the OLS regression results, with columns (1)-(3) incorporating all control variables. Table 3 presents the estimation results for Equation (1). Column (1) does not put into control variables and controls for industry fixed effects and time fixed effects, Column (2) adds some other control variables, such as Top1, IDratio, IsDuality, and Roa. Column (3) further adds other variables, such as Size, Lev, TobinQ, Board, Age, and Ocf. We find the estimated coefficients of ESG are all significant and positive at the 1% level in each column regression. This suggests that independent directors' CPC member status positively improves firms' ESG performance. In terms of the economic significance, taking the results in Column (3) as an instance, for each standard deviation rise in firms' ESG performance, the mean IDparty increases by 1.56% (1.132X0.118/7.784) standard deviations, which is 10% of the mean IDparty 0.44% (1.132X0.118/28.05). It shows that independent directors' CPC member status positively improves firms' ESG performance, thus verifying H1a.

**Table 3:** Baseline regression results.

	Column (1)	Column (2)	Column (3)
	ESG	ESG	ESG
IDparty	1.041***	1.054***	1.132***
	(3.86)	(3.91)	(4.53)
Top1		-0.00498**	-0.0258***
		(-2.15)	(-11.77)
IDratio		2.709***	2.911***
		(5.25)	(5.69)
IsDuality		0.151	0.516**
		(0.64)	(2.36)
Roa		0.153***	0.0836***
		(5.85)	(3.98)
Size			1.814***
			(50.26)
Lev			1.691
			(0.31)
TobinQ			2.587
			(0.47)
Board			0.109***
			(5.54)
Age			-0.0079
			(-1.53)
Ocf			1.131***
			(3.72)
Attention			0.236**
			(2.43)
Big4			-3.442***
			(-28.17)
SOEs			-0.194*
			(-1.73)
_cons	15.78***	15.03***	-19.23***
	(38.96)	(33.55)	(-3.45)
Ind FE	YES	YES	YES
Year FE	YES	YES	YES
N	30629	30629	30629
Adjusted R-squared	0.495	0.496	0.567

This table reports regression results of Eq. (1). ESG is a firm performance score from Bloomberg. The IDparty is a dummy variable of independent directors' CPC member status. The definitions of the other variables are displayed in Table 1. Each regression analysis adds fixed effects for both year and industry. ***, **, and * indicating significance at the 1%, 5%, and 10% levels, respectively.

In terms of the control variables, the coefficients (Size, IDratio, Roe, IsDuality, Board, Attention) are significant and positive, indicating that these variables can improve firms' ESG performance. This result is consistent with previous studies conducted by Chen et al. [12], Naheed et al. [47]. The coefficients (Top1, Big4, SOEs) are significant and negative, these results reflect that these variables can reduce firms' ESG performance, this result is consistent with previous studies conducted by Barko, Cremers, and Renneboog [3], McGuinness, Vieito, and Wang [44]. Other variables have no significance in ESG.

### 4.3 Robustness tests

We also conduct four robustness tests to confirm the validity of the significant positive relationship between independent directors' CPC member status and firms' ESG is robust to the alternative definition of independent variable, alternative definition of dependent variable, lag explained variable, Heckman Two-Stage Regression, instrumental variable, and DID.

#### 4.3.1 Alternative definition of independent variables

To ensure the robustness of our primary conclusions, we perform a substitution of the definition of the independent variable. We measure independent directors' CPC member status using PPC, which is calculated as the percentage of independent directors' CPC member status among party branches. Results are presented in column (1) of Panel A of Table 4, and we find that the positive and significant relationship between independent directors' CPC member status and firms' ESG performance is still robust.

**Table 4:** Robustness tests.

Panel A: Robustness tests				

	Column (1)	Column (2)	Column (3)	Column (4)
	ESG	ESGr	ESG_1	ESG_2

IDparty		0.194**	0.973***	0.938***
		(2.07)	(3.84)	(3.68)
PPC	5.995***			
	(11.85)			
Controls	YES	YES	YES	YES
Constant	-18.192***	-6.286**	-16.74***	-15.56***
	(-3.27)	(-2.14)	(-2.97)	(-2.73)
Ind FE	YES	YES	YES	YES
year FE	YES	YES	YES	YES
N	30,629	6,180	30109	29580
Adjusted R-squared	0.569	0.242	0.555	0.544

Panel B: Endogenous test: Instrumental variable method				

	Column (1)	Column (2)		
	ESG	IDparty		

IDparty	1.051***			
	(4.15)			
IDr		0.834***		
		(11.59)		
Controls	YES	YES		
_cons	-28.48***	-44.56***		
	(-5.06)	(-7.71)		
Ind FE	YES	YES		
Year FE	YES	YES		
Cragg-Donald Wald F		4544.897		
Sargan		0		
N	30629	30629		
Adjusted R-squared	0.556	0.558		

Panel C Endogenous test: Heckman Two-Stage Regression				

	First step	Second step		
	IDparty	ESG		

Controls	YES	YES		
Partyrr	2.182***			
	(23.23)			
IDparty		2.457***		
		(7.50)		
IMR		-0.913***		
		(-6.64)		
_cons	-8.494**	-56.91***		
	(-2.06)	(-7.88)		
Ind FE	YES	YES		
Year FE	YES	YES		
N	30629	30629		
Pseudo R2	0.0125			
Adjusted R-squared		0.272		

This table reports the results of the robustness test. Panel A provides the results of robustness tests. Panel B provides the results of the endogenous test, and using 2SLS. Panel C provides the results of the endogenous test, and using Heckman. PPC is the percentage of independent directors' CPC member status among party branches as an alternative explanatory variable. ESGr is a firm performance score from RANKINS. ESG_1 and ESG_2 respectively lagged one period and lagged two periods. IDr is an instrumental variable. Partyrr is the average number of Communist Party of China members in a firm. The definitions of the other variables are displayed in Table 1. Each regression analysis adds fixed effects for both year and industry.***, **, and * indicating significance at the 1%, 5%, and 10% levels, respectively.

#### 4.3.2 Alternative definition of dependent variables

This paper adopted other ESG databases from RANKINS to test robustness. RKS ESG Ratings is the authoritative third-party rating agency for China's sustainable development. We perform the new dependent variable (ESGr) in the benchmark model. Results are presented in column (2) of Panel A of Table 4, and our analysis reveals that the H1a is still valid.

#### 4.3.3 Lagging explained variable

Lagging explained variables is a way to solve the reverse causality problem, we lag the firms' ESG performance for one period and two periods, and regard them as the explained variables to regress the model. Baseline regression suggests that independent directors' CPC member status is positively correlated with firms' ESG performance, but it may be the result of companies with good firms' ESG performance being more willing and able to choose the members of CPC to serve independent directors, which brings about a reverse causality problem. The lagging period is not easily affected by the positive influence in the firms' ESG performance. Columns (2)-(3) of Panel A of Table 4 show the regression results, which show that the conclusion remains robust.

#### 4.3.4 IV-2SLS

Certain omitted variables and potential reverse causality are important factors that contribute to the unrobust of our findings. Endogeneity presents a potential limitation in our research, as previously highlighted. To mitigate potential endogenous issues, we use the two-stage least squares method (2SLS). We construct instrumental variables for the independent directors' CPC member status. An effective instrumental variable should correlate to the independent variable but be exogenous to the dependent variable.

According to Ain et al. [2], we use the average value of all independent directors' CPC member states that are located in the same province and year to construct the instrumental of IDparty (IDr). For the instrumental variables, we employed a two-stage least squares (2SLS) methodology using IDparty (IDr) as the instrument to assess the impact of independent directors' CPC membership status on firms' ESG performance. Specifically, we firstly use IDr to predict the value of IDparty, and then we use the predicted values from the first-stage regression to explain the impact of independent directors' CPC member status on firms' ESG performance. Meanwhile, we find the results in Panel B of Table 4 are consistent with our primary hypothesis: IDparty exhibits a significant and positive relationship with firms' ESG performance.

Results obtained using the instrumental 2SLS method are shown in the Panel B of Table 4. Column (1) presents the first-stage regression results with the CPC-embeddedness in independent directors as the dependent variables to test the correlation of the instrument variable (IDr). Other control variables are consistent with the baseline regression Table 4, year and industry fixed effects are controlled. The coefficient of IDr is positive and significant at 1% level, suggesting that it has a strong relevance with IDparty. We further conduct the weak identification test (Cragg-Donald F-test statistics) and find that the F-Statistics is 4544.897, and the result of Sargan is 0.000, suggesting that our instrumental variable is not weak instrument. Column (2) shows the regression results of the second stage. Similar to what we observed in the OLS regression, the coefficient on IDparty remains positive and significant.

#### 4.3.5 Heckman two-stage regression

One distinctive feature of Heckman's two-stage regression is its incorporation of an instrumental variable. Instrumental variables are those that affect the dependent variable only through their correlation with other variables. Utilizing an instrumental variable is also considered an effective approach to addressing endogeneity issues, particularly those arising from omitted variables. [27]. Following prior studies, we employ one instrumental variable, the average number of Communist Party of China members (Partyrr). “Our second instrumental variable is grounded in principles that address simultaneity and reverse causality. [27], [26]. We contend that incorporating instrumental variables will not diminish the strength of the relationship between independent directors with CPC member status with firms' ESG performance.

Three conclusions can be drawn from an ideal Heckman's two-stage regression: Although the independent variable is statistically significantly related to the dependent variable and the instrumental variable is significantly correlated with the relevant variables, the MIR does not exhibit a significant association. The results of the Heckman two-step regression are presented in Part C of Table 4.

In column (1) of Panel C, We observed a significant positive correlation between Partyrr and IDparty, both in the first stage (coef. = 2.182, t = 23.23). This result confirms that the average number of CPC member status is an important determinant for the independent director with CPC member status.

The result of second stage is represented in column (2), shown in Panel C. We found a significant positive association between IDparty and the firms' ESG performance. Therefore, we conclude that our initial regression results remain robust, especially concerning the omitted variable Partyrr. While our IMR variable shows a significant correlation with firms' ESG performance, both in the second (coef.=-0.913, t=-6.64). We can infer from this conclusion that the endogeneity problem does not entirely exclude our result. IMR is subsequently added as a control variable to retest in the regression model's second stage, which lessens endogeneity. The conclusions are validated by the coefficient significance of IDparty, which aligns with the previously mentioned results.

#### 4.3.6 DID

Independent directors as an important member of corporate governance, the appointment and departure of independent directors is a significant event for firms and has an important impact on firms' strategic decisions. We further research the effect of the appointment and departure of independent directors with CPC member status on firms' ESG performance. First, we select the firms appointed independent directors with CPC member status from the beginning to the final departure. Then, we use the control variables as a standard for matching to select firms that do not have independent directors from the start. Additionally, we also select the firms that start out with no independent directors with CPC member status and end up with independent directors with CPC member status, and using the control variables as a standard for matching to select firms that do not have independent directors from the start. Finally, we utilize the DID to estimate the effect of the appointment and departure of independent directors with CPC member status in firms' ESG performance.

The results of the DID in Panel A and Panel B of Table 5. The difference-in-differences estimation regression results reported that the appointment of independent directors with CPC member status can significantly improve firms' ESG performance, and the departure of independent directors with CPC member status can significantly decrease firms' ESG performance.

**Table 5:** DID.

Panel A: Departure of the independent director with CPC member status (VS No independent director with CPC member status)				

Outcome	ESG	S.Err.	|*t*|	*P* > |*t*|

Before				
Control	-85.441			
Treated	-87.009			
Diff(T-C)	-1.568	0.081	-19.4	0.000***
After				
Control	-83.041			
Treated	-87.55			
Diff(T-C)	-4.509	0.737	6.12	0.000***
Diff	-2.941	0.741	3.97	0.000***

Panel B: Appointment of the independent director with CPC member status (VS No independent director with CPC member status)				

Outcome	ESG	S.Err.	|*t*|	*P* > |*t*|

Before				
Control	-85.3			
Treated	-86.773			
Diff(T-C)	-1.473	0.305	-4.83	0.000***
After				
Control	-86.827			
Treated	-82.282			
Diff(T-C)	4.544	0.741	6.13	0.000***
Diff	6.017	0.801	7.51	0.000***

This table reports the results of DID. Panel A provides the results of the Departure of the independent director with CPC member status VS No independent director with CPC member status. Panel B provides the results of the Appointment of the independent director with CPC member status VS No independent director with CPC member status. The definitions of the other variables are displayed in Table 1. Each regression analysis adds fixed effects for both year and industry. ***, **, and * indicating significance at the 1%, 5%, and 10% levels, respectively.

Endogeneity remains one of the most challenging issues in empirical research and cannot be entirely eliminated by any empirical test, we use four approaches to mitigate the endogeneity problem and find that our results remain on hold.

## 5 Further tests

### 5.1 Mechanism test

The preceding analysis reveals that the CPC membership status of independent directors exerts a positive and causal influence on the firms' ESG performance, but the mechanism is still ambiguous. We propose a scene that which independent directors' CPC member status attends board meetings, and propose a hypothesis that if the promoting effect of CPC-embeddedness on firms' ESG performance is caused by the frequency of board meetings, the hypothesis should be pronounced in this scene.

Based on the agency cost theory of board governance, we know that the board acts as the central authority in the organizational structure—serving as the ‘nexus’ in a nexus of contracts or the ‘mediating hierarchy’ in a team production model. [19]. Its principal job was to ensure that neither management nor shareholders extracted private benefits at the expense of the other [53]. The board's decisions on what to measure, value, and closely monitor throughout the organization's lifespan. In a world where ESG concerns are becoming ever more critical, the stakes have never been higher. Board independence and diversity substantially improve ESG disclosure practices across all sectors, largely driven by regulatory requirements [32], [19].

A few studies provide evidence that managers and directors are recommended to attach ESG [5]. Based on stakeholder theory, increased board independence mitigates conflicts of interest among stakeholders, fostering management practices that enhance long-term value and promote greater transparency. Consequently, many scholars contend that boards with a higher proportion of independent directors are more inclined to foster enhanced voluntary disclosure and promote greater engagement in CSR investments. On the other hand, returnee directors may have greater reputation concerns about firms' ESG performance and, therefore, monitor management to raise ESG/CSR behavior. Therefore, it is extremely unclear how directors with CPC member status might affect their firms' ESG performance. Furthermore, board meetings promote the exchange of information and perspectives among directors, thereby refining the decision-making process and better aligning with stakeholder expectations in a dynamic business environment.

Referring to the mainstream literature [32], we take stepwise regression to test whether the frequency of board meetings attended by independent directors' CPC is an effective mediating mechanism. Specific procedures are described as follows:

(2) $$ESG_{i,t}=\alpha_{0}+\vartheta_{2}IDparty_{i,t}+\beta_{2}controls_{i,t}+Ind_{i}+Year_{t}+\varepsilon_{i,t}$$

(3) $$BM_{i,t}=\alpha_{0}+\vartheta_{1}IDparty_{i,t}+\beta_{2}controls_{i,t}+Ind_{i}+Year_{t}+\varepsilon_{i,t}$$

(4) $$ESG_{i,t}=\alpha_{0}+\vartheta_{2}IDparty_{i,t}+\omega_{3}BM_{i,t}+\beta_{2}controls_{i,t}+Ind_{i}+Year_{t}+\varepsilon_{i,t}$$

In the above Equation (2) to Equation (4), $BM_{i,t}$ is a mediating variable that symbolizes the frequency of board meetings for firms i in year t, and the rest of the corresponding variables are the same in the Equation (1). We collect the frequency of board meetings (BM) from the China Stock Market Accounting Research Database (CSMAR) to construct the BM. The results of the mechanism test shown in Table 6, we find that the coefficients of IDparty and BM are positive and significant, showing that the board meeting is a channel to influence the role of independent directors with CPC member status. According to Fogel, Ma, and Morck [21] and Burns, Kapalczynski, and Wald [9], directors hold a vital role in corporate governance, they not only offer strategic advice to managerial teams and supervise their actions in firm decision-making. The importance of directors is manifested in the board meetings, where directors exchange ideas and insights. With the increase in the frequency of board meetings, interactions and understanding among directors have also been strengthened, the supervisory and advisory functions of independent directors can also be effectively utilized as a result. Independent directors with CPC member status as members of the board of directors, the ideology of the Communist Party of China can also be transmitted through the attendance of independent directors at board meetings. Therefore, the board meeting is an important mediation mechanism to affect the effect of independent directors' CPC member status.

**Table 6:** Mechanism test.

VARIABLES	Column (1)	Column (2)	Column (3)
	ESG	BM	ESG
IDparty	1.051***	0.246*	1.035***
	(4.15)	(1.23)	(4.09)
BM			0.0650***
			(9.01)
Controls	YES	YES	YES
_cons	-28.48***	-13.95***	-27.59***
	(-5.06)	(-3.13)	(-4.90)
Ind FE	YES	YES	YES
Year FE	YES	YES	YES
N	30629	30626	30626
Adjusted R-squared	0.556	0.199	0.557

This table shows the mechanism test of CPC embeddedness on independent directors and firms' ESG performance. ESG is a firm performance score from Bloomberg. BM is the number of board meeting. The IDparty is a dummy variable of independent directors' CPC member status. All the other control variables are the same as those in Table 3 and the definitions of the other variables are displayed in Table 1. Each regression analysis adds fixed effects for both year and industry. ***, **, and * indicating significance at the 1%, 5%, and 10% levels, respectively.

### 5.2 Moderator effect analysis

Different from the listed firms in Europe and America, the corporate governance system of Chinese listed firms is influenced by the Chinese political system [70], [34]. A distinctive feature of many developing countries is that the closer linkages between political and economic systems compared to Western systems [49], [11].

From these perspectives, firms' ESG activities may be moderated by independent directors' pre-existing political linkages. Such as whether independent directors serve as representatives in the People's Congress or as members of the Chinese People's Political Consultative Conference (CPPCC), and whether independent directors are members of the Federation of Industry and Commerce (FICCI)). Therefore, we use the administrative linkages and associative linkages as two proxies for the pre-existing political linkages. When firms have various resources to grow in a consequence of the pre-existing political linkages, they may not pay attention to the focus of stakeholders and may neglect ESG activities, thereby the effect of the independent directors' CPC member status may not work. We use cross-sectional tests to examine the effect of CPC-embedded independent directors with pre-existing political linkages that are negatively correlated with firms' ESG performance. Specific procedures are described as follows:

(5) $$ESG_{i,t}=\alpha_{0}+\vartheta_{3}IDparty_{i,t}+\beta_{2}controls_{i,t}+Ind_{i}+Year_{t}+\varepsilon_{i,t}$$

(6) $$BM_{i,t}=\alpha_{0}+\vartheta_{4}CP_{i,t}+\beta_{2}controls_{i,t}+Ind_{i}+Year_{t}+\varepsilon_{i,t}$$

(7) $$ESG_{i,t}=\alpha_{0}+\vartheta_{3}IDparty_{i,t}+\omega_{3}IDparty_{i,t}⁎CP_{i,t}+\beta_{2}controls_{i,t}+Ind_{i}+Year_{t}+\varepsilon_{i,t}$$

In the above Equation (5) to Equation (7), $CP_{i,t}$ are respectively as status of Consultative and Polity for firms i in year t, and the rest of the corresponding variables are the same in the Equation (1).

The findings regarding the moderation effects are detailed in Table 7. The regression results reported that the coefficients of interaction terms (IDpartyConsultative and IDpartyPolity) are negatively and significantly at the 1% level, with values less than 0, which demonstrates that the influence of CPC-embeddedness is pronounced in firms with fewer pre-existing political linkages. Therefore, pre-existing political linkages can moderate the effect of CPC-embeddedness in independent directors on firms' ESG performance.

**Table 7:** Moderator effect analysis.

VARIABLES	Column (1)	Column (2)	Column (3)	Column (4)	Column (5)	Column (6)
Consultative		0.308*	0.419**			
		(1.67)	(2.23)			
IDparty	0.037***	1.039***	1.251***	-0.006	1.054***	1.162***
	(4.69)	(4.11)	(4.8)	(-1.08)	(4.16)	(4.53)
IDpartyxConsultative			-3.554***			
			(-3.33)			
Polity					0.513*	0.537*
					(1.86)	(1.94)
IDpartyxPolity						-4.222***
						(-2.46)
Controls	YES	YES	YES	YES	YES	YES
Constant	-0.052	-28.466***	-28.583***	0.048	-28.507***	-28.500***
	(-0.30)	(-5.06)	(-5.08)	(0.41)	(-5.06)	(-5.06)
Ind FE	YES	YES	YES	YES	YES	YES
Year FE	YES	YES	YES	YES	YES	YES
N	30,629	30,629	30,629	30,629	30,629	30,629
Adjusted R-squared	0.028	0.556	0.556	0.021	0.556	0.556

This table shows the moderating test of CPC embeddedness on independent directors and firms' ESG performance. Panel A is the moderating effect of association linkage and administrative linkage. ESG is a firm performance score from Bloomberg. The IDparty is a dummy variable of independent directors' CPC member status. All the other control variables are the same as those in Table 3 and the definitions of the other variables are displayed in Table 1. Each regression analysis adds fixed effects for both year and industry. ***, **, and * indicating significance at the 1%, 5%, and 10% levels, respectively.

### 5.3 Motives analysis

Previous research has emphasized the advantages that political linkages can bring to firms, such as tax incentives [11], bank loans [10], and governmental subsidies [58]. Beyond these findings, we probe deeper into the underlying motivations of independent directors of the Communist Party of China to promote firms' ESG performance. We discuss these motivations across three fronts: (1) Bank loans; (2) Government subsidies; and (3) Tax incentives. We use cross-sectional tests to examine the motivation of CPC embedded in independent directors to affect firms' ESG performance.

Results of the subsample tests are reported in columns (1)-(3) of Table 8. Column (1) reveals that the coefficient for the interaction term IDpartyESG is significantly positive at 10%, the result suggests that independent directors who are CPC members have stronger motivation to obtain bank loans. Independent directors are able to utilize their political status to alleviate the financial constraints. However, column (2) reveals that the coefficient for the interaction term IDpartyESG is significantly negative at 10%, the result suggests that independent directors who are CPC members will decrease the motivation to obtain tax incentives. On the one hand, it is of an ex-post nature. Tax incentives may not be immediately available for improving production processes or implementing sustainable management. As a result, independent directors' CPC member status may not have sufficient incentives to obtain tax incentives. On the other hand, social pressure. Due to stakeholders' attention to the firms' ESG performance, independent directors who are members of CPC may be more willing to take on additional financial burdens, even if it means giving up certain tax incentives. For government subsidies, independent directors who are CPC members have no corresponding relationship with firms' ESG performance. Taking all these considerations into account, the paper finds that the motivation for independent directors with CCP membership to enhance firms' ESG performance is to obtain bank loans and alleviate tax burdens.

**Table 8:** Motives analysis.

VARIABLES	Column (1)	Column (2)	Column (3)
	Loan	Tax	GSubsidy
ESG	0.00875***	0.000849***	0.0189***
	(3.67)	(4.66)	(13.72)
IDparty	-1.013**	0.0567	-0.332
	(-2.03)	(1.39)	(-1.16)
IDpartyxESG	0.0295*	-0.00236*	0.00369
	(1.95)	(-1.87)	(0.43)
_cons	-7.820***	0.910***	-5.955***
	(-3.50)	(5.1)	(-4.65)
Ind FE	YES	YES	YES
Year FE	YES	YES	YES
N	20238	30629	20075
Adjusted R-squared	0.168	0.22	0.499

This table shows the motives of firms' ESG activities. ESG is a firm performance score from Bloomberg. Loan is Bank loans. Tax is Tax incentives. GSubsidy is Government subsidies. All the other control variables are the same as those in Table 3 and the definitions of the other variables are displayed in Table 1. Each regression analysis adds fixed effects for both year and industry. ***, **, and * indicating significance at the 1%, 5%, and 10% levels, respectively.

### 5.4 Further analysis

#### 5.4.1 External supervision

The Big Four accounting firms have strong professional capabilities and global recognition, Big Four accounting firms play an important role in enhancing corporate disclosure and internal controls, and it helps investors alleviate the information asymmetry. Various scholars think the Big Four accounting firms have higher audit quality, reputation, and market recognition, so they have a stronger motivation to strengthen the firm's external supervision to maintain their reputation [28]. If the firms are supervised by Big Four accounting firms, the function of independent directors may be influenced. Thus, we use the Big Four accounting firms as the external supervision to test the role of independent directors' CPC member status in firms' ESG performance.

Panel A of Table 9 shows that the coefficient is significant and positive at 1% level in the subsample of non-big Four accounting firms, and the coefficient is not significant in the subsample of Big Four accounting firms. The results suggest that independent directors' CPC member status is more effective for governance in firms without external supervision.

**Table 9:** Further analysis.

	Panel A		Panel B		Panel C		Panel D
	Big4	Non-Big4	Low-Attention	High-Attention	SOEs	Non-SOEs	FSGP
IDparty	-0.432	1.419***	0.548	1.142***	0.695***	0.878*	0.963***
	(-0.38)	(5.72)	(0.8)	(4.19)	(2.62)	(1.8)	(3.49)
IDpartyxPolicy							3.885***
							(4.44)
Controls	YES	YES	YES	YES	YES	YES	YES
_cons	-268.1***	-22.29***	-45.734**	-26.445***	-28.50***	-2.991	-31.21***
	(-5.58)	(-4.11)	(-2.56)	(-4.45)	(-5.06)	(-0.22)	(-4.95)
IndFE	YES	YES	YES	YES	YES	YES	YES
YearFE	YES	YES	YES	YES	YES	YES	YES
N	2214	28415	3,686	26,943	27495	3134	3116
Adjusted R-squared	0.673	0.566	0.534	0.558	0.54	0.675	0.565

This table shows the further analysis results. ESG is a firm performance score from Bloomberg. Panel A reports the results of external supervision. Panel B reports the results of the attention level of independent directors who are CPC members. Panel C reports the results of ownership nature. Panel D reports the results of impact of the policy of “full and strict governance over the Party”. The IDparty is a dummy variable of independent directors' CPC member status. All the other control variables are the same as those in Table 3 and the definitions of the other variables are displayed in Table 1. Each regression analysis adds fixed effects for both year and industry. ***, **, and * indicating significance at the 1%, 5%, and 10% levels, respectively.

#### 5.4.2 Attention level

Based on behavioral theory, we know that a person's consciousness impacts their behaviors, therefore attention plays an importance in their behaviors. The attention of independent directors who are CPC members has great importance in firms' making-decisions. Those who serve as independent directors are often influential people in the industry and are highly specialized. Independent directors hold concurrent positions in multiple firms [51]. When they serve as independent directors within more firms, the busier they are and the less able they are to advise on the firms' business decisions, firms' ESG performance will further be poor. We define the subsample of below-average number of concurrent appointments as high attention and the subsample of above-average number of concurrent appointments as low attention.

Panel B of Table 9 shows that the coefficient is significant and positive in 1% level in the subsample of high attention, and the coefficient is not significant in the subsample of low attention. The results suggest that independent directors who have CPC member status can improve firms' ESG performance under high attention, otherwise, the effect is invalid.

#### 5.4.3 Ownership nature

Different ownership structures in firms lead to varying firms' strategies [67], so we then sort our sample by the shares' nature of property rights, results are displayed in Panel C of Table 9, where presents the results in the state-owned firms (SOEs), and non-state-owned firms (Non-SOEs). We find that the coefficients are all positive and significant, and the coefficients are larger in the Non-SOEs subsample than the corresponding coefficients in the SOEs subsample. Indicating that the effect of CPC-embeddedness on independent directors is more pronounced in SOEs. Previous studies indicate that SOEs have a stronger awareness of social responsibilities than Non-SOEs [69]. Therefore, SOEs have a less impactful driving effect of independent directors' CPC member status on firms' ESG performance compared to non-SOEs.

#### 5.4.4 “Full and strict governance over the party”

Since the policy of “full and strict governance over the Party”^4^ has been published, party members' behaviors have received wide attention from others. For firms, the policy not only decreases the probability of corruption and rent-seeking but also cultivates a favorable business environment. Thus, we think the strategy of “full and strict governance over the Party” may influence the decision style, from aggressive to robust, it is good for firms' ESG performance.

Panel D in Table 9 shows that the coefficient for the interaction term IDpartyrPolicy significantly positive at 1%, and the coefficient increases from 0.695 to 3.885, which suggests that the policy of “full and strict governance over the Party” has significantly strengthened the CPC-embeddedness.

## 6 Conclusions

This paper focuses on Chinese listed firms over the period 2012–2021 as the research subjects and explores the independent directors' CPC member status and its impact on firms' ESG performance. We implemented a set of robustness analyses, and the results remained consistent despite variations in the definition of the independent variable, replacing the dependent variable, and lagging the explained variable, Heckman Two-Stages, IV-2SLS, in line with DID. The mechanism test suggests that board meeting is an effective mechanism to transform the influence of the CPC into corporate governance, further improving firms' ESG performance. In addition, we investigate the motivations of independent directors with CPC member status to improve firms' ESG performance from three key areas: bank loans, government subsidies, and tax. We find independent directors who are members of the Communist Party of China (CPC) have incentives to obtain bank loans and reduce tax burdens to improve the firms' ESG performance. Finally, we also investigate the external supervision, attention level, policy of “full and strict governance over the Party”, and ownership nature. Finding that CPC members in independent directors serve as alternative supervisors for the firms' ESG performance, the effect of independent directors' CPC member status is in the subsample of high attention, the more evident the promotion role of the CPC in firms' ESG performance when independent directors have the fewer concurrent roles, the effect of independent directors' CPC member status in SOEs is stronger than Non-SOEs, the effect of independent directors' CPC member status has been enhanced after the implementation the policy of “full and strict governance over the Party”.

Our findings not only deepen our understanding of the implications of firms' ESG performance but also offer critical insights for policymakers, investors, auditors, and economic analysts. Based on the political connection theory, independent directors who have CPC member status serve as crucial communication bridges and goal coordinators between the government and firms. They facilitate the transmission of the government's public governance goals to firms, thereby encouraging them to enhance their ESG performance. From the perspective of affiliation with the ruling Communist Party, our study reveals the independent directors' Party status as a significant driver of ESG. According to the upper echelons theory, CPC member status can influence a firm's decision-making, thus firms want to improve corporate governance by focusing on independent directors' political status. Besides, the paper provides some advice for stakeholders. Investors need to pay attention to the independent directors' background of party members, policymakers can add political factors into incentives and penalties for ESG performance. Auditors and economic analysts can know the proportion of independent directors with CPC member status and thus provide them with a certain dimension to consider for an accurate evaluation of the firms.

This study has some limitations. For financial performance, we only investigate the relationship between independent directors' CPC member status and firms' ESG performance but do not provide the financial impact of firms' ESG performance. Furthermore, there may be several mechanisms for independent directors' CPC member status to promote firms' ESG performance. However, due to data limitations, we can not further explore other mechanisms. Furthermore, from a research standpoint, our paper enhances the comprehension of the embeddedness of CPC in listed firms and the role of independent directors in emerging markets. Future research may look more into how this improved ESG performance affects firms' financial performance. Additionally, examining the effectiveness of ESG investments advocated by independent directors with CPC membership would provide valuable insights.

## Funding

1. 2022 Philosophy and social sciences research project of 10.13039/100012554Hubei Provincial Department of Education (No. 22Q105). 2. Research Funding of 10.13039/501100008960Wuhan Polytechnic University (No. 2022RZ005). 3. 2023 Wuhan Federation of Social Sciences (No. 2023039).

## CRediT authorship contribution statement

**Fang Jia:** Writing – review & editing, Writing – original draft, Visualization, Validation, Funding acquisition, Formal analysis, Data curation, Conceptualization. **Xuerong Li:** Writing – review & editing, Writing – original draft, Visualization, Validation, Supervision, Software, Resources, Project administration, Formal analysis, Data curation, Conceptualization. **Yan Gao:** Validation, Supervision, Software, Resources, Project administration, Methodology.

## Declaration of Competing Interest

The authors declare that they have no known competing financial interests or personal relationships that could have appeared to influence the work reported in this paper.

## Data Availability

The name of the repository and the accession number: (figshare), the accession number: 10.6084/m9.figshare.25734327.

## Footnotes

1 Party branch (jiceng dangzuzhi) is the smallest unit of the Chinese Communist Party (CCP) at the grassroots level. One of the unique characteristics of CCP is its “mass line” (qunzhong luxian) (Han 2015). In other words, CCP relies on numerous party branches to exercise its control over the state, economy and society in China.

2 On August 21, 2001, the China Securities Regulatory Commission post the “Guiding Opinions on the Introduction of Independent Directors for Listed firms.

3 The Communist Party of China (CPC) issued Rule No. 18 on October 10,2013, the Rule 18 was issued to clarify rules and disciplinary measures within the party to enhance compliance and accountability. Rule No. 18 mandates that party and government officials above certain ranks, either incumbent or retired within the past three years, are prohibited from holding any part-time or full-time positions in any companies.

4 In 2016, the Sixth Plenary Session of the 18th Central Committee of the Communist Party of China formally proposed the important deployment of “comprehensively strengthening the Party's leadership and Party building,” emphasizing the need for “full and strict governance over the Party”. This initiative has attracted widespread attention both within and outside the Party and is regarded as one of the significant measures by the Communist Party of China to enhance self-purification, self-improvement, self-renewal, and self-elevation.
